# Linear biocompatible glyco-polyamidoamines as dual action mode virus infection inhibitors with potential as broad-spectrum microbicides for sexually transmitted diseases

**DOI:** 10.1038/srep33393

**Published:** 2016-09-19

**Authors:** Nicolò Mauro, Paolo Ferruti, Elisabetta Ranucci, Amedea Manfredi, Angela Berzi, Mario Clerici, Valeria Cagno, David Lembo, Alessandro Palmioli, Sara Sattin

**Affiliations:** 1 Dipartimento di Chimica, Università degli Studi di Milano, via C. Golgi 19, 20133 Milan, Italy; 2 Consorzio Interuniversitario di Scienza e Tecnologia dei Materiali, via G. Giusti 9, 56121 Firenze, Italy; 3 Department of Biomedical and Clinical Sciences “Sacco”, University of Milan, via G. B. Grassi 74, 20157 Milan, Italy; 4 Department of Medical, Surgical and Transplants Physiopathology, University of Milan, via Fratelli Cervi 93, 20090 Segrate, Milan, and Don C. Gnocchi Foundation IRCCS, Via Capecelatro 66, 20148 Milan, Italy; 5 Dipartimento di Scienze Cliniche e Biologiche, Università di Torino, Azienda Ospedaliero Universitaria S. Luigi Gonzaga, via Regione Gonzole 10, 10043 Orbassano, Torino, Italy

## Abstract

The initial steps of viral infections are mediated by interactions between viral proteins and cellular receptors. Blocking the latter with high-affinity ligands may inhibit infection. DC-SIGN, a C-type lectin receptor expressed by immature dendritic cells and macrophages, mediates human immunodeficiency virus (HIV) infection by recognizing mannose clusters on the HIV-1 gp120 envelope glycoprotein. Mannosylated glycodendrimers act as HIV entry inhibitors thanks to their ability to block this receptor. Previously, an amphoteric, but prevailingly cationic polyamidoamine named AGMA1 proved effective as infection inhibitor for several heparan sulfate proteoglycan-dependent viruses, such as human papilloma virus HPV-16 and herpes simplex virus HSV-2. An amphoteric, but prevailingly anionic PAA named ISA23 proved inactive. It was speculated that the substitution of mannosylated units for a limited percentage of AGMA1 repeating units, while imparting anti-HIV activity, would preserve the fundamentals of its HPV-16 and HSV-2 infection inhibitory activity. In this work, four biocompatible linear PAAs carrying different amounts of mannosyl-triazolyl pendants, Man-ISA_7_, Man-ISA_14_, Man-AGMA_6.5_ and Man-AGMA_14.5_, were prepared by reaction of 2-(azidoethyl)-α-D-mannopyranoside and differently propargyl-substituted AGMA1 and ISA23. All mannosylated PAAs inhibited HIV infection. Both Man-AGMA_6.5_ and Man-AGMA_14.5_ maintained the HPV-16 and HSV-2 activity of the parent polymer, proving broad-spectrum, dual action mode virus infection inhibitors.

Viral entry, the first stage of viral infection, is mediated by multiple interactions between viral attachment proteins and specific cellular receptors^1^. Blocking these cell receptors with high-affinity, selective ligands is a strategy for devising antiviral agents, and multivalency is a powerful chemical tool to address this issue^2^,^3^,^4^,^5^. Dendritic Cell-Specific Intercellular adhesion molecule-3-Grabbing Non-integrin (DC-SIGN), a C-type lectin receptor primarily expressed on the surface of immature dendritic cells (DCs) and macrophages, acts as recognition molecule in the modulation of the innate immune system response. DC-SIGN also plays a prominent role in the early infection stages of several human pathogens, including, among others, human immunodeficiency virus (HIV)^6^,^7^. DC-SIGN binds to glycan ligands found on both human cells and bacterial or parasitic pathogens, with a recognized specificity for those containing mannose and fucose^8^. It specifically recognizes high-mannose clusters on the gp120 envelope glycoprotein of HIV-1^9^, establishes strong multipoint interactions and promotes viral internalization and trans-infection of the T-lymphocytes facilitating HIV dissemination^10^. The lectin is presented at the surface of dendritic cells as a tetramer, with four identical carbohydrate recognition domains (CRDs) that interact in a Ca^2+^ dependent manner with their glycan targets. Each CRD contains a sugar-binding Ca^2+^ site and the sites are separated by about 4 nm^11^. Synthetic multivalent ligands with DC-SIGN affinity have the potential to block viral entry^12^,^13^. Mannosylated glycodendrimers^14^ glycopolymers^9^ and glycosylated nanoparticles^15^,^16^ have been developed to this target. These materials showed good activities only when a high number of mannose units was displayed. At lower loadings, more complex but also more active ligands (*i.e.* oligomannosides of variable size) were preferred.

Recently, it has been demonstrated that a tetravalent presentation of a pseudo-trimannoside interacts with DC-SIGN and blocks HIV-1 infection both in cellular and in human cervical explanted models and that glycodendrimers bearing multiple copies of pseudo-mannosyl groups exert HIV and Dengue inhibitory activity^17^,^18^,^19^.

Many viruses, bacteria, and parasites express adhesins that facilitate initial cell attachment and subsequent infection by binding to cell surface Heparan Sulfate ProteoGlycans (HSPGs)^20^. Polycations may prevent virus adsorption by electrostatically interacting either with the negatively charged cell membrane, or with the envelope of lipid-enveloped viruses. For instance, Eudragit E 100, a cationic poly(acrylic ester), was active against several lipid-enveloped viruses^21^. Cell pre-incubation with polyethylenimine reduced human papilloma virus (HPV) and human cytomegalovirus (HCMV) infections^22^. Poly-L-lysine, poly-L-arginine, poly-L-histidine and cationic polysaccharides were significantly active against herpes simplex virus (HSV)^23^. Unfortunately, many synthetic polycations active against HSPG-dependent viruses are significantly cytotoxic. This is not the case of linear polyamidoamines (PAAs), a family of mostly water-soluble polymers obtained by Michael-type polyaddition of prim- or bis-sec-amines with bisacrylamides, that can be planned to be biocompatible and biodegradable^24^. Two amphoteric PAAs, named ISA23^25^,^26^ and AGMA1^27^,^28^,^29^ whose chemical structures are reported in Fig. 1, proved exceptionally cytobiocompatible.

Their isoelectric points were 5.2 (ISA23) and 10.3 (AGMA1). At pH 7.4 they carried on average −0.50 and +0.55 excess charge per unit, respectively. In infection inhibition tests, carried out *in vitro* on a panel of HSPG-dependent viruses, namely HSV, HPV, human cytomegalovirus (HCMV) and respiratory syncytial virus (RSV), AGMA1 was remarkably active^30^,^31^. The AGMA1 activity was attributed to its ability to interact with HSPGs^32^. However, AGMA1 did not inhibit HIV infection. In all cases, ISA23 proved completely inactive owing to its prevailingly anionic charge, not apt to provide significant interactions with HSPGs.

It was speculated that the substitution of mannosylated units for a limited percentage of AGMA1 repeating units, while imparting anti-HIV activity, would essentially preserve the fundamentals of its HPV-16 and HSV-2 infection inhibitory activity. If verified, this hypothesis opened the way to dual-action-mode broad-spectrum polymeric antiviral agents. In this line, partially mannosylated ISA23 and AGMA1 were prepared as potential DC-SIGN antagonists, and tested as HIV-1 trans infection inhibitors. The reason for choosing two PAAs of different physico-chemical and antiviral properties was to discriminate between a possible anti-HIV activity due solely to the mannosyl pendants, and an anti-HIV activity benefitting from a synergistic effect by the polymer chain. Such an effect was hardly predictable for mannosylated ISA23, but could not be excluded for mannosylated AGMA1, since the establishment of HIV interactions with cell membrane HSPGs is an early step of the infection.

## Results and Discussion

### Synthesis

**Figure 2:**
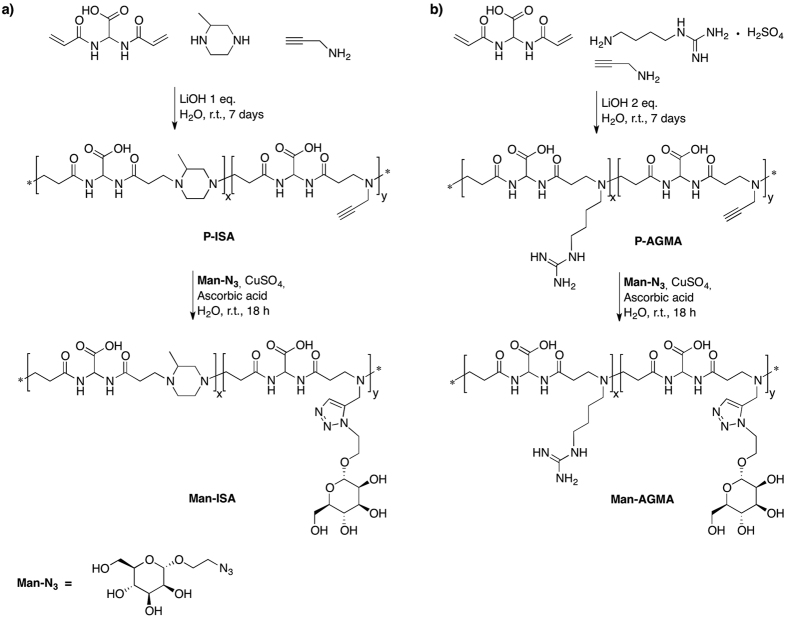
Synthesis of (**a**) Man-ISA and (**b**) Man-AGMA polymers. Two batches of each polymer were prepared with different content of propargylamine bearing repeating units: P-ISA_7_ and P-ISA_14_ (7.0 and 14.0%); P-AGMA_6.5_ and P-AGMA_14.5_ (6.5 and 14.5%). The corresponding mannosylated polymers had the same mannosyl unit content.

ISA23 and AGMA1 are normally prepared by stepwise polyaddition of 2,2-bis(acrylamido)acetic acid with, respectively, 2-methylpiperazine and 4-amminobutylguanidine (agmatine). In this work, mannosylated ISA23 and AGMA1 were prepared in two steps (details provided in the Supplementary Information). In the first step, two modified ISA23 (P-ISA) and two modified AGMA1 (P-AGMA) carrying propargyl pendants were prepared by substituting 10- or 20% (on a molar basis) propargylamine for 2-methylpiperazine or agmatine in the polymerization recipes (Fig. 2). The crude products were purified by ultrafiltration through a membrane with nominal cut-off 3000. In the purified products, the actual percentages on a molar basis of propargylamine-bearing repeating units were, as assessed by ^1^H NMR, 7.0% and 14.0% (ISA23); 6.5% and 14.5% (AGMA1). Accordingly, the final products were named P-ISA_7_, P-ISA_14_, P-AGMA_6.5_ and P-AGMA_14.5_. There is no reason to believe that the propargyl units were orderly inserted in the polymer chains, since stepwise polyaddition reactions of monomer mixtures with similar reactivity normally lead to random copolymers. In the second step, a copper acetate-catalyzed alkyne-azide cycloaddition (“click” reaction) was performed between the propargylamine-modified PAAs and 2-(azidoethyl)-α-D-mannopyranoside, leading to the corresponding mannosylated ISA23 (Man-ISA_7_, Man-ISA_14_) and AGMA1 (Man-AGMA_6.5_ and Man-AGMA_14.5_).

The copper ions were removed from the final products by carefully acidifying to pH 3.5 with hydrochloric acid and ultrafiltering through a membrane with nominal cut-off 3000. The residual copper content, as determined by atomic absorption was in all cases <0.01% on a w/w basis. The retained fractions were finally retrieved by lyophilization. The representative NMR spectra of P-ISA_7_, Man-ISA_7_, P-AGMA_6.5_ and Man-AGMA_6.5_ are reported in Figs S1–S4. The quantitative assessment of the percentage of substitution of propargylamine units was performed from the relative intensities of the integrals of the C**H**_2_ α to the triple bond (4.18 ppm) and C**H**COOH (5.58 or 5.63 ppm for P-ISA and P-AGMA, respectively) peaks. The crosspeak signal of this CH_2_ in the HSQC spectra disappeared in the mannosylated products (compare Figs S1–S4). This was assumed as an indication that the reaction approached completion.

### Biossays

#### HIV infection inhibitory activity

**Figure 3:**
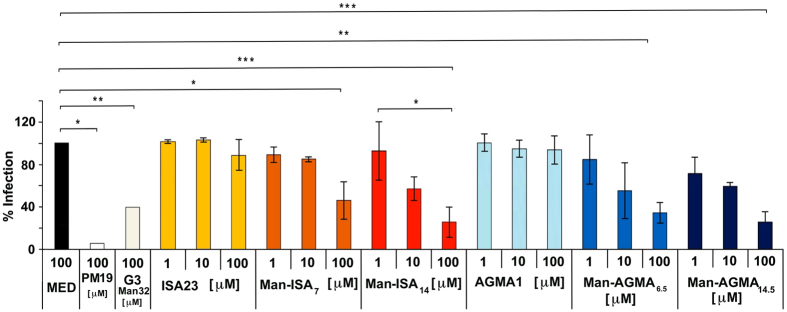
HIV trans infection levels. HIV-1 productive infection was measured as amount of p24 protein in the co-culture supernatants. Experiments were performed on CD4+ T lymphocytes isolated from 3 different healthy donors. Data represent the percent HIV-1 infection following polymer treatment in comparison with the untreated control (MED, set as 100% of HIV infection). For Man-AGMA and Man-ISA samples, concentrations refer to the mannosylated units. In case of plain ISA23 and AGMA1, the same w/v concentrations of Man-ISA_7_ and Man-AGMA_6.5_ were used, respectively. Values represent the mean ± SD. Statistical analysis was performed using one-way ANOVA followed by the Bonferroni’s post hoc test. *P < 0.05, **P < 0.01, ***P < 0.001.

The ability of Man-ISA and Man-AGMA samples to inhibit HIV-1 trans infection of CD4+ T lymphocytes was evaluated exploiting the B-THP-1/DC-SIGN cells as a model of dendritic cells (DCs). This model is well-established and has been repeatedly shown to depend on DC-SIGN^10^,^33^,^34^ by using BTHP-1 cells not expressing DC-SIGN as a negative control. Trans infection of CD4+ T lymphocytes was monitored by enzyme-linked immunosorbent assay (ELISA) for the HIV-1 protein p24^17^. The results are reported in Fig. 3, where concentrations are referred to the mannose units and shown as percent inhibition relative to the culture medium (negative) control, which is assigned 100% infection value. The previously described PM19, a highly potent hexavalent glycomimetic, is used as a positive control (compound 13.4 in ref. 17). Of note, the efficacy of monovalent mannose is too low to be measured in the millimolar concentration range used in these tests. Results show for all samples a mannosyl-concentration-dependent HIV infection inhibiting activity. The same tests confirmed the inefficacy in this respect of plain ISA23 and AGMA1.

It may be noticed that the mannosylated PAAs reported here were significantly active against HIV notwithstanding their molecular architecture lacked a well-defined steric arrangement of the saccharide groups, since the propargylamine units (hence the mannosyl pendants in the final product) were expected to be randomly distributed along the polymer chain, as it is the rule in stepwise polyaddition polymers. In mannosylated PAAs, the mannosyl groups were attached as pendants to linear, mobile polymer chains that in solution could assume multiple conformations.

The molecular weights and, per macromolecule, the average number of repeating units and the average number of mannosyl pendants are reported in Table 1.

With the exception of Man-ISA_14,_ the molecular weights were not very high and, notably, the number of mannosyl pendants per macromolecule was modest. None of these factors played a role in determining the antiviral activity, which solely depended on the mannosyl group concentration in the culture medium. As evident from Fig. 3, in fact, the amount of mannosylated PAA needed to reach a given level of anti-HIV activity was in all cases inversely proportional to its mannosylation degree. In other words, the specific activity of the mannosyl pendants attached to both ISA23 and AGMA1 was independent of their frequency along the PAA chain as well as of the PAA’s nature, molecular weight and net average charge in the culturing medium. It had been previously found that amphoteric PAAs, different from non-amphoteric ones, give molecular aggregates in aqueous media owing to ionic interactions^35^.

In the present case, the ability of forming clusters, combined with the intrinsic conformational mobility of the polymer chains, probably allowed several mannosyl groups attached to either the same or different macromolecules to cooperate in establishing strong interactions with the lectin receptors.

No synergistic effect on the anti-HIV activity of the AGMA1 carrier was demonstrated. Finally, it may be noticed that the HIV infection inhibitory activity of Man-ISA_14_ and Man-AGMA_14.5_ was comparable to that of G3Man_32_, a previously reported Bolton mannosylated dendrimer with valence 32^36^. Although higher activity dendrimers have been reported^19^, they all carry monovalent ligands intrinsically more active than mannose. The anti-HIV activity of Man-AGMA and Man-ISA samples was not due to cytotoxic effect on the host DCs since, as shown in Fig. S5, no appreciable cell toxicity was observed in the concentration range adopted in the infection inhibitory activity tests. This corresponds to the cytobiocompatibility of plain ISA23 and AGMA1. The introduction of mannosyl-triazolyl pendants was apparently inconsequential in this respect.

#### HPV and HSV infection inhibitory activity

**Figure 4:**
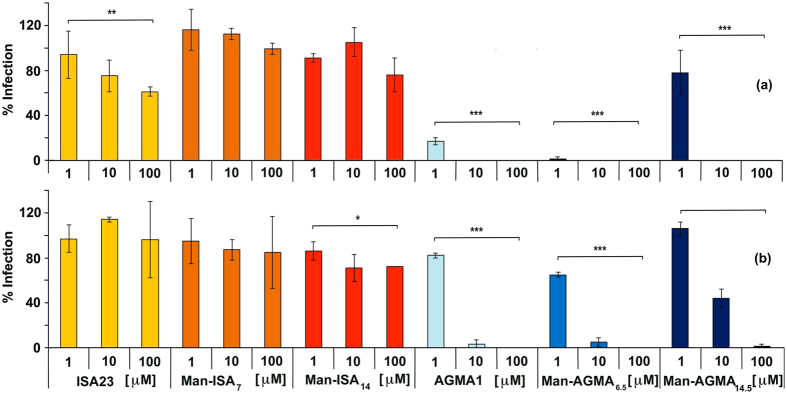
(**a**) HPV-16 and **(b)** HSV-2 infection inhibitory activity. Data represent the percent infection following polymer treatment in comparison with the untreated control. For Man-AGMA and Man-ISA samples, concentrations refer to the mannosylated units. In case of plain ISA23 and AGMA1, the same w/v concentrations of Man-ISA_7_ and Man-AGMA_6.5_ were used, respectively. Values represent the mean ± SD. Statistical analysis was performed using two-way ANOVA followed by the Bonferroni’s post hoc test. *P < 0.05, **P < 0.01, ***P < 0.001.

As mentioned, AGMA1 had previously proven endowed with intrinsic antiviral activity towards several HSPG dependent viruses, including HPV and HSV, and represents a good positive control of inhibition. The experiments were performed on the oncogenic HPV-16 and HSV-2, both sexually transmitted and often associated to HIV infections^37^. As shown in Fig. 4, neither ISA23 nor its mannosylated derivatives exerted any HPV-16 and HSV-2 infection inhibitory activity. On the opposite, both Man-AGMA_6.5_ and Man-AGMA_14.5_ were significantly active against both viruses. Interestingly, at low concentration, Man-AGMA_14.5_ was less active, likely because the mannosylated units had replaced an equal number of guanidine-bearing units that, according to previous studies^31^, are responsible for the AGMA1 anti-HPV and anti-HSV activity. Of note, also in these experiments none of the compounds proved cytotoxic at the tested doses and at the same time-points used for antiviral assays (Figs S6 and S7), further confirming that antiviral activity was not a consequence of cytotoxicity.

## Experimental Section

### Chemistry section

(Detailed information is available in Supplementary Information).

### Biological section

#### HIV assay

##### *In vitro* trans-infection assay of CD4+ T lymphocytes

The assay was performed as previously described^38^,^39^. All experimental protocols were approved by the Institutional Review Board of Vimercate Hospital (Italy). CD4+ T lymphocytes were purified from Peripheral Blood Mononuclear Cells (PBMCs) isolated from buffy coats obtained from healthy volunteers after written informed consent and all methods were carried out in accordance with the approved guidelines. Briefly, B-THP1/DC-SIGN and B-THP1 cells (NIBSC, Potter Bar, UK) were pre-incubated for 30 min with the polymers and then pulsed with 40 TCID_50_ HIV-1 BaL (NIBSC, Potter Bar, UK), without removing the inhibitors for 3 h. After washing, the cells were cultured with the pre-activated CD4+ T lymphocytes for 3 days. HIV p24 concentration in the co-culture supernatants was assessed by ELISA (Express Bio, Thurmont, MD, USA).

##### BTHP1-DC-SIGN Cell viability assay

BTHP1-DC-SIGN cells were incubated with the polymer samples for different time points (3 h and 30 min, 24 h, and 72 h) and then labelled with 7-aminoactinomycin D (7-AAD) (Beckman Coulter, Milan, Italy). Samples were acquired by using a Gallios™ Flow Cytometer and data were analysed with Kaluza^®^ Flow Analysis Software (both from Beckman Coulter). HeLa and Vero cells were seeded at in 96-well plates; the next day, they were treated with serially diluted compounds. After 24 or 72 h of incubation, cell viability was determined using the CellTiter 96 Proliferation Assay Kit (Promega, Madison, WI, USA), according to the manufacturer’s instructions. Absorbances were measured using a Microplate Reader (Model 680, BIORAD) at 490 nm.

#### HPV assay

##### Cell culture

The human cervical carcinoma cell lines HeLa were grown as monolayers in Dulbecco’s modified Eagle’s medium (DMEM) (Gibco-BRL, Gaithersburg, MD) supplemented with heat-inactivated 10% fetal calf serum (FCS; Gibco- BRL). The 293TT cell line, derived from human embryonic kidney cells transformed with the simian virus 40 (SV40) large T antigen, was cultured in the medium described above supplemented with nonessential amino acids. 293TT cells allow high levels of protein to be expressed from vectors containing the SV40 origin due to overreplication of the expression plasmid^40^. African green monkey fibroblastoid kidney cells (Vero, ATCC CCL-81) were grown as monolayers in Eagle’s minimal essential medium (MEM) (Gibco/BRL, Gaithersburg, MD) supplemented with 10% heat inactivated fetal calf serum (FCS) and 1% antibiotic-antimycotic solution (Zell Shield, Minerva Biolabs GmbH, Berlin, Germany).

##### HPV PsV production

Plasmids and 293TT cells used for pseudovirus (PsV) production were kindly provided by John Schiller (National Cancer Institute, Bethesda, MD) or bought at Addgene (Cambridge, MA). Detailed protocols and plasmid maps for this study are reported at http://home.ccr.cancer.gov/lco/pseudovirusproduction.htm. HPV-16 PsVs were produced according to previously described methods^41^. Briefly, 293TT cells were transfected with plasmids expressing the papillomavirus major and minor capsid proteins (L1 and L2, respectively), together with a reporter plasmid expressing the green fluorescent protein (GFP), named pfwB. Capsids were allowed to mature overnight in cell lysate; the clarified supernatant was then loaded on top of a density gradient of 27 to 33 to 39% Optiprep (Sigma-Aldrich, St. Louis, MO) at room temperature for 3 h. The material was centrifuged at 28000 rpm for 18 h at room temperature in an SW41.1 rotor (Beckman Coulter, Inc., Fullerton, CA) and then collected by bottom puncture of the tubes. Fractions were inspected for purity in 10% sodium dodecyl sulfate (SDS)–Tris–glycine gels, titrated on 293TT cells to test for infectivity by SEAP or GFP detection, and then pooled and frozen at −80 °C until needed. The L1 protein content of PsV stocks was determined by comparison with bovine serum albumin standards in Coomassie-stained SDS-polyacrylamide gels^32^.

##### HPV GFP-based assays

HeLa cells were seeded in 96-well plates in 100 ml of DMEM supplemented with 10% FBS. The next day, serial dilutions of compounds were added to preplated cells together with dilutions of PsV stock. After 72 h of incubation at 37 °C fluorescent cells were counted on an inverted Zeiss LSM510 fluorescence microscope.

#### HSV assay

##### Viruses

A clinical isolate of HSV-2 was kindly provided by Professor M. Pistello, University of Pisa, Italy. HSV-2 was propagated and titrated by plaque assay on Vero cells.

##### HSV-2 inhibition assay

The effect of compounds on HSV-2 infection was evaluated by plaque reduction assay. Vero cells were pre-plated 24 h in advance in 24-well plates at a density of 10 × 10^4^ cells. Serial dilutions of compounds and HSV-2 (MOI 0.0005 pfu/cell) were added to cells for 2 h. The virus inoculum was then removed and the cells washed and overlaid with a medium containing 1.2% methylcellulose (Sigma-Aldrich, Milano, Italy). After further incubation at 37 °C for 24 h cells were fixed and stained with 0.1% crystal violet in 20% ethanol and viral plaques counted. The percent of infection was calculated in comparison with the untreated control.

### Statistical Analysis

For infection studies, comparisons between groups were performed using one-way (HIV) or two-way (HPV and HSV) ANOVA followed by a Bonferroni’s Multiple Comparison Test. A P value less than 0.05 was considered statistically significant (*P < 0.05, **P < 0.01, ***P < 0.001). Statistical analysis was performed using GraphPad Prism 5 (GraphPad Software, San Diego, CA, USA).

## Conclusions

In this work, two biocompatible PAAs named ISA23 and AGMA1 functionalised with moderate amounts of mannosyl-triazolyl pendants, Man-ISA_7_, Man-ISA_14_, Man-AGMA_6.5_ and Man-AGMA_14.5_, were successfully prepared by click reaction between 2-(azidoethyl)-α-D-mannopyranoside and partially propargyl-substituted PAA precursors, in turn prepared by substituting 10 or 20% (on a molar basis) propargylamine for 2-methylpiperazine (ISA23) or agmatine (AGMA1) in the reaction recipes. All Man-ISA and Man-AGMA samples proved effective HIV infection inhibitors notwithstanding their molecular architecture lacked a preliminarily defined spatial arrangement of the saccharide groups. Their inhibitory activity was independent of the nature of the PAA carrier, but solely governed be the concentration of mannosyl residues in the culture medium, higher substituted samples reaching the same efficacy at lower polymer concentrations. The antiviral activity could be probably ascribed to the fact that in solution the PAA carriers, having mobile conformations, may form clusters driving several mannosyl residues to establish multiple interactions with the lectin receptors.

AGMA1 had previously proved inactive as HIV infection inhibitor, but very active against several HSPG-dependent viruses. Man-AGMA_6.5_ and Man-AGMA_14.5_ maintained this activity, as tested against the sexually transmitted HPV-16 and HSV-2 viruses. Thus, mannosylation imparted anti-HIV activity to both ISA23 and AGMA1, and preserved the latter’s HPV and HSV inhibitory activity. These results point the final conclusion that Man-AGMA derivatives warrant potential as dual-action-mode, broad-spectrum inhibitors of sexually transmitted viral infections.

As a future development, AGMA1 or ISA23 derivatives carrying different glycomimetic substituents intrinsically endowed with significant activity as DC-SIGN antagonists can be considered.

## Additional Information

**How to cite this article**: Mauro, N. *et al*. Linear biocompatible glyco-polyamidoamines as dual action mode virus infection inhibitors with potential as broad-spectrum microbicides for sexually transmitted diseases. *Sci. Rep.* **6**, 33393; doi: 10.1038/srep33393 (2016).

## Supplementary Material

Supplementary Information

## Figures and Tables

**Figure 1:**
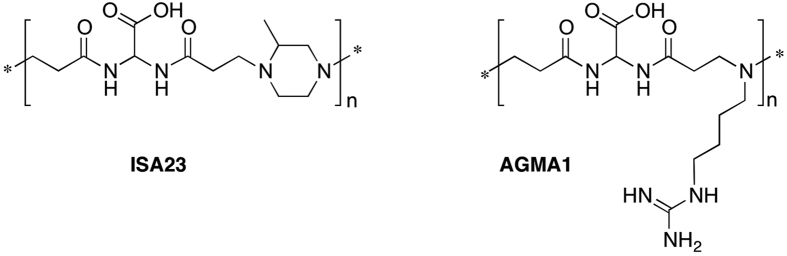
Chemical structures of ISA23 and AGMA1.

**Table 1:** Molecular weight, average number of repeating units and number of mannosyl pendants of mannosylated PAAs.

Sample	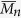	PD	Average N° repeating units per macromolecule	Average N° mannosyl residues per macromolecule
Man-ISA_7_	8700	1.09	28	1.96
Man-ISA_14_	21900	1.44	67	9.38
Man-AGMA_6.5_	7500	1.21	22	1.43
Man-AGMA_14.5_	8100	1.21	23	3.33

## References

[b1] Wickham T. J., Granados R. R., Wood H. A., Hammer D. A. & Shuler M. L. General analysis of receptor-mediated viral attachment to cell surfaces. Biophys. J. 58, 1501–1516 (1990). 2177356 10.1016/S0006-3495(90)82495-4 PMC1281102

[b2] Choi S.-K. Synthetic Multivalent Molecules: Concepts and Biomedical Applications. (John Wiley & Sons: Hoboken, 2004).

[b3] Schmidt S., Wang H., Pussak D., Mosca S. & Hartmann L. Probing multivalency in ligand-receptor-mediated adhesion of soft, biomimetic interfaces. Beilstein J. Org. Chem. 11, 720–729 (2015). 26124875 10.3762/bjoc.11.82 PMC4464160

[b4] Danial M. & Klok H. A. Polymeric anti-HIV therapeutics. Macromol. Biosci. 15, 9–35 (2015). 25185484 10.1002/mabi.201400298

[b5] Bernardi A. . Multivalent glycoconjugates as anti-pathogenic agents. Chemical Society reviews 42, 4709–4727 (2013). 23254759 10.1039/c2cs35408j PMC4399576

[b6] van Kooyk Y. & Geijtenbeek T. B. DC-SIGN: escape mechanism for pathogens. Nat. Rev. Immunol. 3, 697–709 (2003). 12949494 10.1038/nri1182

[b7] Mercer J. & Greber U. F. Virus interactions with endocytic pathways in macrophages and dendritic cells. Trends Microbiol. 21, 380–388 (2013). 23830563 10.1016/j.tim.2013.06.001

[b8] van Liempt E. . Specificity of DC-SIGN for mannose- and fucose-containing glycans. FEBS Lett. 580, 6123–6131 (2006). 17055489 10.1016/j.febslet.2006.10.009

[b9] Becer C. R. . High-Affinity Glycopolymer Binding to Human DC-SIGN and Disruption of DC-SIGN Interactions with HIV Envelope Glycoprotein. J. Am. Chem. Soc. 132, 15130–15132 (2010). 20932025 10.1021/ja1056714 PMC3091610

[b10] Geijtenbeek T. B. H. . DC-SIGN, a Dendritic Cell Specific HIV-1-Binding Protein that Enhances trans-Infection of T Cells. Cell 100, 587–597 (2000). 10721995 10.1016/s0092-8674(00)80694-7

[b11] Tabarani G. . DC-SIGN neck domain is a pH-sensor controlling oligomerization: SAXS and hydrodynamic studies of extracellular domain. The Journal of biological chemistry 284, 21229–21240 (2009). 19502234 10.1074/jbc.M109.021204 PMC2755846

[b12] Anderluh M., Jug G., Svajger U. & Obermajer N. DC-SIGN Antagonists, a Potential New Class of Anti-Infectives. Curr. Med. Chem. 19, 992–1007 (2012). 22257062 10.2174/092986712799320664

[b13] Reina J. J. & Rojo J. Glycodendritic structures: tools to interact with DC-SIGN. Braz. J. Pharm. Sci. 49, 109–124 (2013).

[b14] Sánchez-Navarro M. & Rojo J. Targeting DC-SIGN with carbohydrate multivalent systems. Drug News Perspect 23, 557–572 (2010). 21152451 10.1358/dnp.2010.23.9.1437246

[b15] Martinez-Avila O. . Gold manno-glyconanoparticles: multivalent systems to block HIV-1 gp120 binding to the lectin DC-SIGN. Chem Eur J 15, 9874–9888 (2009). 19681073 10.1002/chem.200900923

[b16] Martinez-Avila O. . Multivalent manno-glyconanoparticles inhibit DC-SIGN-mediated HIV-1 trans-infection of human T cells. Chem Bio Chem. 10, 1806–1809 (2009). 10.1002/cbic.200900294 19565596

[b17] Varga N. . A multivalent inhibitor of the DC-SIGN dependent uptake of HIV-1 and Dengue virus. Biomaterials 35, 4175–4184 (2014). 24508075 10.1016/j.biomaterials.2014.01.014

[b18] Berzi A. . Pseudo-Mannosylated DC-SIGN Ligands as Potential Adjuvants for HIV Vaccines. Viruses 6, 391–403 (2014). 24473338 10.3390/v6020391 PMC3939462

[b19] Ordanini S. . Designing nanomolar antagonists of DC-SIGN-mediated HIV infection: ligand presentation using molecular rods. Chem. Commun. 51, 3816–3819 (2015). 10.1039/c4cc09709b 25648900

[b20] Rostand K. S. & Esko J. D. Microbial adherence to and invasion through proteoglycans. Infect Immun 65, 1–8 (1997). 8975885 10.1128/iai.65.1.1-8.1997 PMC174549

[b21] Alasino R. V. . Amphipathic and Membrane-Destabilizing Properties of the Cationic Acrylate Polymer Eudragit^®^ E100. Macromol Biosci 5, 207–213 (2005). 15768439 10.1002/mabi.200400168

[b22] Spoden G. A. . Polyethylenimine Is a Strong Inhibitor of Human Papillomavirus and Cytomegalovirus Infection. Antimicrob. Agents Ch. 56, 75–82 (2012). 10.1128/AAC.05147-11 PMC3256013 21968369

[b23] Yudovin-Farber I., Gurt I., Hope R., Domb A. J. & Katz E. Inhibition of herpes simplex virus by polyamines. Antivir. Chem. Chemoth. 20, 87–98 (2009). 10.3851/IMP1401 19843979

[b24] Ferruti P. Poly(amidoamine)s: Past, present, and perspectives. J. Polym. Sci. A1 51, 2319–2353 (2013).

[b25] Ferruti P. . Synthesis, characterisation and antitumour activity of platinum(II) complexes of novel functionalised poly(amido amine)s. Macromol. Chem. Phys. 200, 1644–1654 (1999).

[b26] Richardson S., Ferruti P. & Duncan R. Poly(amidoamine)s as Potential Endosomolytic Polymers: Evaluation *in vitro* and Body Distribution in Normal and Tumour-Bearing Animals. J. Drug Target. 6, 391–404 (1999). 10937285 10.3109/10611869908996846

[b27] Franchini J., Ranucci E., Ferruti P., Rossi M. & Cavalli R. Synthesis, Physicochemical Properties, and Preliminary Biological Characterizations of a Novel Amphoteric Agmatine-Based Poly(amidoamine) with RGD-Like Repeating Units. Biomacromolecules 7, 1215–1222 (2006). 16602741 10.1021/bm060054m

[b28] Ferruti P. . Prevailingly Cationic Agmatine-Based Amphoteric Polyamidoamine as a Nontoxic, Nonhemolytic, and “Stealthlike” DNA Complexing Agent and Transfection Promoter. Biomacromolecules 8, 1498–1504 (2007). 17388564 10.1021/bm061126c

[b29] Cavalli R. . Amphoteric Agmatine Containing Polyamidoamines as Carriers for Plasmid DNA *in vitro* and *in vivo* Delivery. Biomacromolecules 11, 2667–2674 (2010). 20815397 10.1021/bm100685t

[b30] Donalisio M. . Agmatine-Containing Poly(amidoamine)s as a Novel Class of Antiviral Macromolecules: Structural Properties and *in vitro* Evaluation of Infectivity Inhibition. Antimicrob. Agents Ch. 58, 6315–6319 (2014). 10.1128/AAC.03420-14 PMC4187951 25092704

[b31] Donalisio M. . The AGMA1 poly(amidoamine) inhibits the infectivity of herpes simplex virus in cell lines, in human cervicovaginal histocultures, and in vaginally infected mice. Biomaterials 85, 40–53 (2016). 26854390 10.1016/j.biomaterials.2016.01.055

[b32] Cagno V. . The Agmatine-Containing Poly(Amidoamine) Polymer AGMA1 Binds Cell Surface Heparan Sulfates and Prevents Attachment of Mucosal Human Papillomaviruses. Antimicrob. Agents Ch. 59, 5250–5259 (2015). 10.1128/AAC.00443-15 PMC4538463 26077258

[b33] Wu L., Martin T. D., Carrington M. & KewalRamani V. N. Raji B cells, misidentified as THP-1 cells, stimulate DC-SIGN-mediated HIV transmission. Virology 318, 17–23 (2004). 14972530 10.1016/j.virol.2003.09.028

[b34] Sattin S. . Inhibition of DC-SIGN-mediated HIV infection by a linear trimannoside mimic in a tetravalent presentation. ACS Chem. Biol. 5, 301–312 (2010). 20085340 10.1021/cb900216e

[b35] Mendichi R., Ferruti P. & Malgesini B. Evidence of aggregation in dilute solution of amphoteric poly(amido-amine)s by size exclusion chromatography. Biomed. Chromatogr. 19, 196–201 (2005). 15627285 10.1002/bmc.434

[b36] Tabarani G. . Mannose hyperbranched dendritic polymers interact with clustered organization of DC-SIGN and inhibit gp120 binding. FEBS Lett. 580, 2402–2408 (2006). 16616922 10.1016/j.febslet.2006.03.061

[b37] Konopnicki D., De Wit S. & Clumeck N. HPV and HIV Coinfection. A Complex Interaction Resulting in Epidemiological, Clinical and Therapeutic Implications. Future Virol. 8, 903–915 (2012).

[b38] Sattin S. . Inhibition of DC-SIGN-Mediated HIV Infection by a Linear Trimannoside Mimic in a Tetravalent Presentation. ACS Chemical Biology 5, 301–312 (2010). 20085340 10.1021/cb900216e

[b39] Berzi A. . A glycomimetic compound inhibits DC-SIGN-mediated HIV infection in cellular and cervical explant models. Aids 26, 127–137 (2012). 22045343 10.1097/QAD.0b013e32834e1567

[b40] Buck C. B., Pastrana D. V., Lowy D. R. & Schiller J. T. Efficient Intracellular Assembly of Papillomaviral Vectors. J.Virol. 78, 751–757 (2004). 14694107 10.1128/JVI.78.2.751-757.2004 PMC368835

[b41] Buck C., Pastrana D., Lowy D. & Schiller J. In Human Papillomaviruses Vol. 119 Methods in Molecular Medicine (eds Davy Clare & Doorbar John) Ch. 32, 445–462 (Humana Press, 2006). 10.1385/1-59259-982-6:445 16350417

