# Supplementary material for: Linear biocompatible glyco-polyamidoamines as dual action mode virus infection inhibitors with potential as broad-spectrum microbicides for sexually transmitted diseases
Source: Sci Rep. 2016 Sep 19;6:33393. doi: 10.1038/srep33393 (PMC5027566; doi:10.1038/srep33393)
Supplement: Supplementary Information [file srep33393-s1.pdf]

Supplementary Information

Linear biocompatible glyco-polyamidoamines as dual action mode virus infection inhibitors with potential as broad-spectrum microbicides for sexually transmitted diseases

Nicolò Mauro[1], Paolo Ferruti[1,2], Elisabetta Ranucci[1], Amedea Manfredi[1], Angela Berzi[3], Mario Clerici[4], Valeria Cagno[5], David Lembo[5], Alessandro Palmioli[1], Sara Sattin[1]

[1] Dipartimento di Chimica, Università degli Studi di Milano, via C. Golgi 19, 20133 Milan, Italy

[2] Consorzio Interuniversitario di Scienza e Tecnologia dei Materiali, via G. Giusti 9, 56121 Firenze, Italy

[3] Department of Biomedical and Clinical Sciences "Sacco", University of Milan, via G. B. Grassi 74, 20157 Milan, Italy

[4] Department of Medical, Surgical and Transplants Physiopathology, University of Milan, via Fratelli Cervi, 93, 20090 Segrate, Milan, and Don C. Gnocchi Foundation IRCCS, Via Capecelatro 66, 20148 Milan, Italy

[5] Dipartimento di Scienze Cliniche e Biologiche, Università di Torino, Azienda Ospedaliero Universitaria S. Luigi Gonzaga, via Regione Gonzole 10, 10043 Orbassano, Torino, Italy

Materials and instrumental methods

2,2-Bis(acrylamido)acetic acid (95%) was synthesized as previously described;[1] lithium hydroxide monohydrate (99%), 2-methylpiperazine (95%), hydrochloric acid (37%), sodium ascorbate (99%), copper sulfate pentahydrate (99%), and agmatine sulfate (97%) were purchased from Sigma-Aldrich and used as received. Propargylamine (98%) was purchased from Aldrich and purified by distillation at atmospheric pressure, collecting the main fraction at 78-80 °C. Ultrapure water was obtained by using a Millipore-Merck Milli-Q® system.

Atomic absorption analyses were performed with a Perkin Elmer Atomic Absorption Spectrometer PinAAcle900T equipped with AS 900 flame autosampler.

^{1}H-NMR spectra were recorded at 400 MHz and ^{13}C-NMR spectra were recorded at 100 MHz, using a Bruker Avance 400 instrument. Chemical shifts (δ) are reported in ppm using residual solvent signals from deuterated solvents as references.[2] Signals in ^{1}H and ^{13}C NMR spectra were

assigned with the aid of two dimensional HSQC spectra. For some signals in ^{1}H-NMR spectra, the coupling patterns were reported as (broad) multiplets due to high order coupling or signal overlap. Size exclusion chromatography (SEC) traces were obtained using a Knauer Pump 1000 equipped with a Knauer Autosampler 3800, TKSgel G4000 PW, and G3000 PW TosoHaas columns connected in series, light scattering/viscometer Viscotek 270 Dual Detector and a refractive index detector Waters model 2410. The mobile phase was a 0.1 M Tris buffer pH 8.1 ± 0.05 with 0.2 M sodium chloride. The sample concentration was 20 mg/mL and the flow rate 1 mL/ min.

Chemical synthesis and characterization

(2-Azidoethyl)-α-D-Mannopyranoside.

This compound was prepared as previously described.[3]

^{1}H-NMR (400 MHz, D_2O) δ: 4.93 (d, $J_{1\text{-}2}$ = 1.6 Hz, 1H. H_1), 3.99 (dd, $J_{2\text{-}3}$ = 3.4, $J_{1\text{-}2}$ = 1.6 Hz, 1H, H_2), 3.97 – 3.88 (m, 2H, H_{6a}, H_{7a}), 3.85 (dd, $J_{3\text{-}4}$ = 9.3, $J_{2\text{-}3}$ =3.4 Hz, 1H, H_3), 3.80 – 3.64 (m, 4H, H_4, H_5, H_{6b}, H_{7b}), 3.56 (ddd, J_{gem} = 13.6, $J_{8a\text{-}7a}$ = 6.7, $J_{8a\text{-}7b}$ = 3.2 Hz, 1H, H_{8a}), 3.50 (ddd, J_{gem} = 13.6, $J_{8b\text{-}7b}$ = 6.3, $J_{8b\text{-}7a}$ =3.3 Hz, 1H, H_{8b}).

^{13}C-NMR (100 MHz, D_2O) δ: 99.8 (C_1), 72.9 (C_4), 70.4 (C_3), 69.9 (C_2), 66.7 (C_5), 66.3 (C_7), 60.9 (C_6), 50.2 (C_8).

ISA23-co-BACPropargylamine 90:10 (P-ISA_7)

2,2-Bis(acrylamido)acetic acid (BAC) (1.000 g, 4.75 mmol) was dissolved in a lithium hydroxide monohydrate (0.201 g, 4.75 mmol) aqueous solution (1.57 mL water). 2-Methylpiperazine (0.452 g, 4.28 mmol) and propargylamine (0.027 g, 0.48 mmol) were added to the reactive mixture under vigorous stirring. The reaction was kept 7 days under nitrogen atmosphere with occasional stirring. After this time morpholine (0.1 g) was added, and the resultant mixture was kept under the same conditions for further 2 days and then acidified to pH = 4 with dilute hydrochloric acid, ultrafiltered through a membrane with nominal molecular weight cut-off 3000 and finally freeze-dried.

Yield: 53%. $\overline{M}_n$ =8300, $\overline{M}_w / \overline{M}_n$ = 1.10, hydrodynamic radius (R_h) = 2.83 nm.

^{1}H-NMR (400 MHz, D_2O) δ: 1.39 (d, C**H**$_3$CH, *J* = 5.6 Hz), 2.67-2.82 (m, C**H**$_2$CONH), 2.83-2.99 (m, NCH$_2$C**H**N), 3.00-3.35 (bm, NC**H$_2$**CHN, ≡C**H**), 3.40-3.74 (m, NC**H$_2$**C**H$_2$**N), 4.18 (bs, C**H**$_2$C≡), 5.54-5.66 (m, C**H**COOH).

^{13}C-NMR (100 MHz, D_2O) δ: 172.9 (COOH), 172.1, 171.6, 171.2 (NHCO), 80.6 (≡**C**H), 70.8(≡C), 58.1 (**C**HCOOH), 56.2, 55.1 (**C**H$_2$CONH), 52.4 (NCH$_2$), 49.7, 49.3 (N**C**H$_2$CH$_2$N), 48.5 (NCH$_2$**C**H$_2$N), 48.2 (NCH$_2$**C**HN), 42.5 (**C**H$_2$C≡), 30.2, 29.7, 29.1 (N**C**H$_2$CHN), 13.9 (**C**H$_3$CH).

ISA23-co-BACMannopyranoside 90:10 (Man-ISA_7)

P-ISA_7 (200 mg) was dissolved in oxygen free water (2 mL) and the solution maintained under nitrogen atmosphere. An aqueous solution of (2-azidoethyl)-α-D-mannopyranoside (11.96 mg, 0.048 mmol), sodium ascorbate (13.5 mg, 0.07 mmol) and copper sulfate pentahydrate (8.5 mg, 0.034 mmol) in water (0.5 mL) was flushed with nitrogen and added to the P-ISA_7 solution. After 18 h the reacting mixture was acidified to pH 3.5 and the solution maintained at this pH value while ultrafiltering through a membrane with nominal molecular weight cut-off 3000 until complete disappearance in the filtered solution of the blue coloring due to the copper/ethylenediamine complex ions upon adding a few ethylenediamine drops. The product was freeze-dried and retrieved

as a white powder. Its copper content, as determined by atomic absorption analysis, was found <0.01%.

Yield: 43%. $\overline{M}_n$ = 8700, $\bar{M}_w/\bar{M}_n$ = 1.09, hydrodynamic radius (R_h) = 2.79 nm.

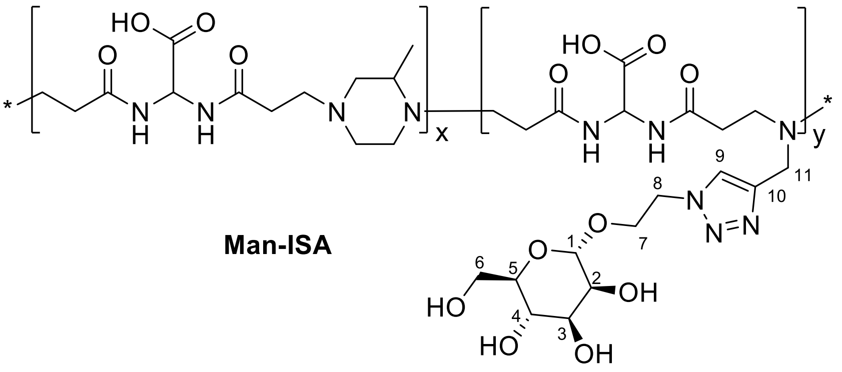

Man-ISA

^{1}H-NMR (400 MHz, D_2O) δ: 1.35 (d, J = 5.8 Hz, C**H_3**CH *pol*), 2.56-3.61 (bm, C**H_2**CONH *pol*, C**H_2**N *pol*, NC**H_2**CH ring, NC**H_2**C**H_2**N ring *pol*, H_4 Man), 3.61–3.75 (m, H_3, H_5, H_{6a} Man), 3.79 (bd, J = 11.6 Hz, H_{6b} Man), 3.90 (bs, H_2 Man), 3.95-4.03 (bm, H_{7a}), 4.13-4.22 (bm, H_{7b}), 4.58 (bs, H_{11}), 4.78 (H_8 from HSQC), 4.86 (H_1 Man *HSQC*), 5.50-5.67 (m, C**H**-COOH and C**H**-COO$^-$ *pol*), 8.39 (s, NC**H**C Triaz), 8.73-8.83 (bm, NH), 8.83-8.92 (bm, NH).

^{13}C-NMR (100 MHz, D_2O) δ: 173.0, 172.8, 172.2, 167.9 (**C**OOH, **C**ONH), 171.4 (N**C**CH Triaz), 128.2 (NC**C**H Triaz), 99.6 (C_1 Man), 73.0 (C_4 Man), 70.5 (C_3 Man), 69.9 (C_2 Man), 66.5 (C_5 Man), 65.3 (C_7 Man), 60.8 (C_6 Man), 58.2 (**C**HCOOH), 56.0, 52.3, 49.9, 49.1, 48.03 (N**C**H_2CH_2N, NCH$_2$**C**HN, N**C**H$_2$CH$_2$CO, N**C**HCH$_3$), 50.4 (C_8 Man), 46.8 (C_{11}),), 30.9, 30.1 (**C**H$_2$CONH), 14.3 (**C**H$_3$CH).

ISA23-co-BACPropargylamine 80:20 (P-ISA$_{14}$)

The reaction was carried out from BAC (1,000 g, 4.75 mmol), lithium hydroxide monohydrate (0.201 g, 4.75 mmol), 2-methylpiperazine (0.401 g, 3.80 mmol), propargylamine (0.053 g, 0.95 mmol) and water (1.57 mL), following the same procedure as for P-ISA$_7$.

Yield: 72%. $\overline{M}_n$ = 24100, $\bar{M}_w / \bar{M}_n$ = 1.35, R_h = 4.45 nm.

ISA23-co-BACMannopyranoside 80:20 (Man-ISA$_{14}$)

The reaction was carried out from P-ISA$_{14}$ (200 mg), (2-azidoethyl)-α-D-mannopyranoside (23.92 mg, 0.096 mmol), sodium ascorbate (27.0 mg, 0.14 mmol), copper sulfate pentahydrate (17.0 mg, 0.068 mmol) and water (0.8 mL), by following the same procedure as for Man-ISA$_7$.

Yield: 52%. $\overline{M}_n$=21900, $\bar{M}_w / \bar{M}_n$ =1.16, R_h = 4.17 nm.

AGMA1-co-BACPropargylamine 90:10 (P-AGMA$_{6.5}$)

The reaction was carried out following the same procedure as for P-ISA$_7$ from BAC (1,000 g, 4.75 mmol), lithium hydroxide monohydrate (0.383 g, 9.03 mmol), agmatine sulfate (1.006 g, 4.28 mmol), propargylamine (0.027 g, 0.48 mmol) and water (1.57 mL).

Yield: 57%. $\overline{M}_n$ = 6700, $\bar{M}_w / \bar{M}_n$ = 1.54, R_h = 2.14 nm.

^{1}H-NMR (400 MHz, D$_2$O) δ: 1.60-1.72 (bm, NCH$_2$C**H$_2$**CH$_2$CH$_2$NH), 1.76-1.90 (bm, NCH$_2$CH$_2$C**H$_2$**CH$_2$NH), 2.78-2.97 (bm, C**H$_2$**CONH), 3.17 (bs, ≡C**H**), 3.25 (m, NC**H$_2$**CH$_2$CH$_2$C**H$_2$**NH), 3.42-3.67 (bm, NC**H$_2$**CH$_2$CO), 4.18 (bs, NC**H$_2$**C≡CH), 5.63 (s, C**H**-COOH)

^{13}C-NMR (100 MHz, D$_2$O) δ: 172.8 (**C**OOH), 171.3 (**C**ONH), 156.8 (**C**=NH), 80.6 (≡**C**H), 70.9 (≡**C**), 58.0 (**C**HCOOH), 52.7 (N**C**H$_2$CH$_2$CH$_2$**C**H$_2$NH), 49.7, 49.2 (NH**C**H$_2$CH$_2$CONH), 42.3 (**C**H$_2$C≡), 40.5 (N**C**H$_2$CH$_2$CH$_2$**C**H$_2$NH), 29.0 (**C**H$_2$CONH), 25.0 (NCH$_2$**C**H$_2$CH$_2$CH$_2$NH), 20.5 (NCH$_2$CH$_2$**C**H$_2$CH$_2$NH).

AGMA1-co-BACMannopyranoside 90:10 (Man-AGMA$_{6.5}$)

The reaction was carried out from P-AGMA$_{6.5}$ (200 mg), (2-azidoethyl)-α-D-mannopyranoside (10.0 mg, 0.040 mmol), sodium ascorbate (12.2 mg, 0.062 mmol), copper sulfate pentahydrate (17.0 mg, 0.068 mmol) and water (0.5 mL), following the same procedure as for Man-ISA$_7$.

Yield: 56%. $\overline{M}_n$ = 7100, $\bar{M}_w / \bar{M}_n$ = 1.21, R_h = 2.03.

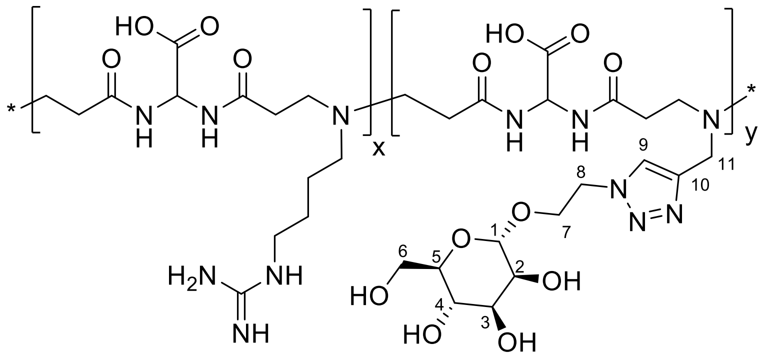

Man-AGMA

^{1}H-NMR (400 MHz, D_2O) δ: 1.65-1.79 (bm, $NCH_2C\mathbf{H_2}CH_2CH_2NH$), 1.80-1.93 (bm, $NCH_2CH_2C\mathbf{H_2}CH_2NH$), 2.81-2.95 (bm, $C\mathbf{H_2}CONH$), 3.15 (H_4 Man *HSQC*), 3.21-3.36 (bm, $NC\mathbf{H_2}CH_2CH_2C\mathbf{H_2}NH$), 3.41 (s, H_{11}, $NC\mathbf{H_2}CH_2CO$), 3.45-3.60 (bm, $NC\mathbf{H_2}CH_2CO$), 3.65 (H_5 Man *HSQC*), 3.70 (H_3 Man *HSQC*), 3.71-3.85 (H_{6a}, H_{6b} Man *HSQC*), 3.93 (H_2 Man *HSQC*), 4.02, 4.19 (H_{7a} H_{7b} Man linker *HSQC*), 4.76 (H_8 Man linker *HSQC*), 4.87 (H_1 Man *HSQC*), 5.70 (bs, C**H**-COOH), 8.23 (C**H** Triaz *HSQC*).

^{13}C-NMR (100 MHz, D_2O) δ: 173.0 (**C**OOH), 171.6 (**C**ONH), 167.9 (NCH**C**, Triaz) 156.9 (**C**=NH), 126.7 (N**C**HC Triaz *HSQC*), 99.7 (C_1 Man), 73.0 (C_4 Man), 70.5 (C_3 Man), 70.0 (C_2 Man), 66.5 (C_5 Man), 65.5 (C_7 Man linker), 60.8 (C_6 Man), 58.2 (**C**HCOOH), 52.7 ($N\mathbf{C}H_2CH_2CH_2\mathbf{C}H_2NH$), 50.2 ($\mathbf{C}_8$ linker), 49.2 ($NH\mathbf{C}H_2CH_2CONH$), 43.4 (C_{11}), 40.5 ($N\mathbf{C}H_2CH_2CH_2\mathbf{C}H_2NH$), 29.3 ($\mathbf{C}H_2CONH$), 25.1 ($NCH_2\mathbf{C}H_2CH_2CH_2NH$), 20.8 ($NCH_2CH_2\mathbf{C}H_2CH_2NH$).

AGMA1-co-BACPropargylamine 80:20 (P-AGMA$_{14.5}$)

The reaction was carried out from 2,2-bis(acrylamido)acetic acid (1,000 g, 4.75 mmol), lithium hydroxide monohydrate (0.362 g, 8.55 mmol), agmatine sulfate (0.894 g, 3.80 mmol), propargylamine (0.053 g, 0.95 mmol) and water (1.57 mL), following the same procedure as for P-ISA$_7$.

Yield: 73%. $\overline{M}_n$ = 8100, $\bar{M}_w / \bar{M}_n$ = 1.93, R_h = 2.57 nm.

AGMA1-co-BACMannopyranoside 80:20 (Man-AGMA$_{14.5}$)

The reaction was carried out from P-AGMA$_{14.5}$ (200 mg), (2-azidoethyl)-α-D-mannopyranoside (20.0 mg, 0.080 mmol), sodium ascorbate (24.4 mg, 0.124 mmol), copper sulfate pentahydrate (34.0 mg, 0.136 mmol) and water (0.8 mL), by following the same procedure as for Man-ISA$_7$. Yield: 48%. $\overline{M}_n$ = 9500, $\bar{M}_w / \bar{M}_n$ = 1.21, R_h = 2.38 nm.

Figure S1: ^{1}H-^{13}C hetero-correlated and ^{13}C spectra of P-ISA$_7$ (D_2O)

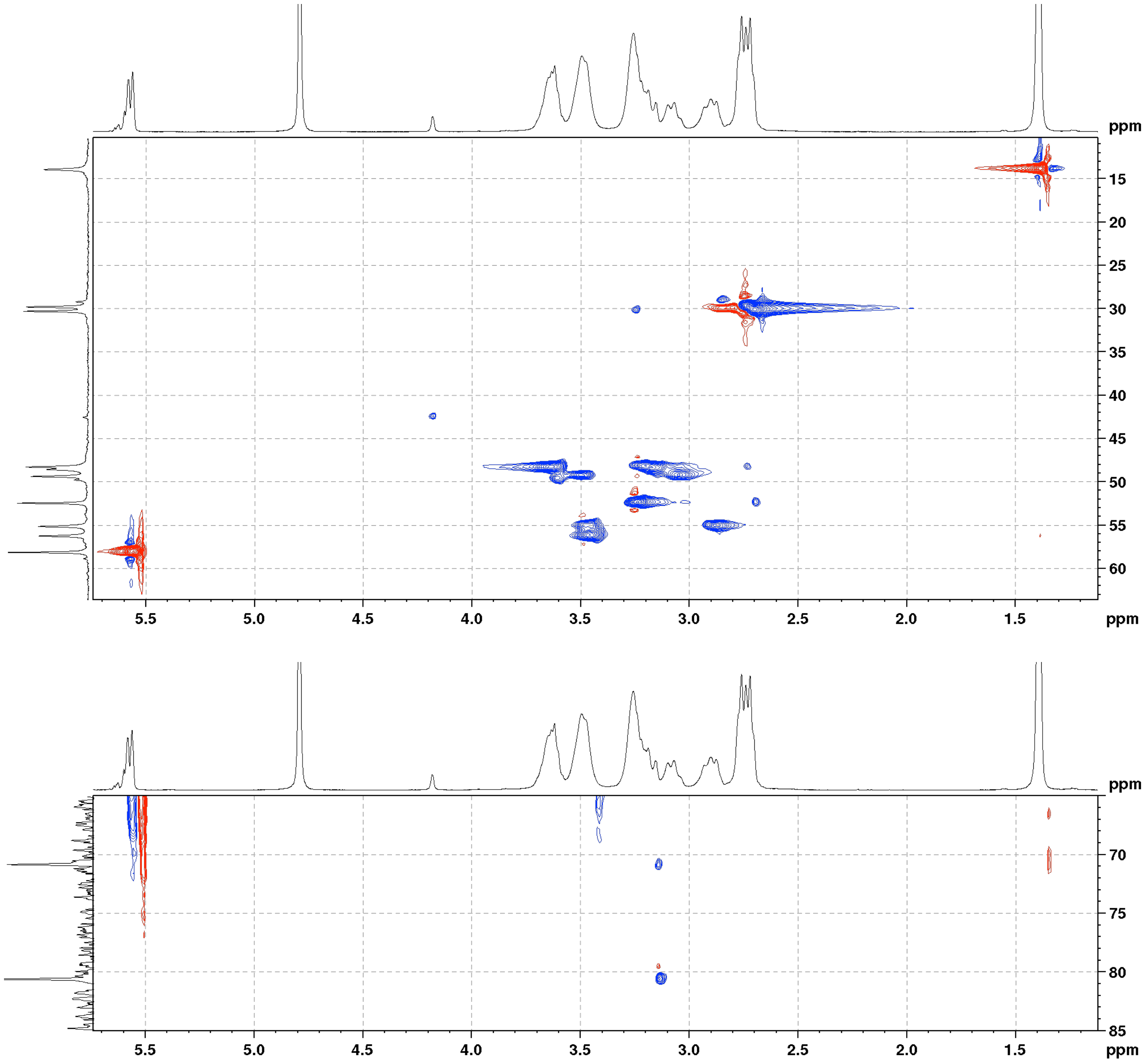

^{13}C-NMR

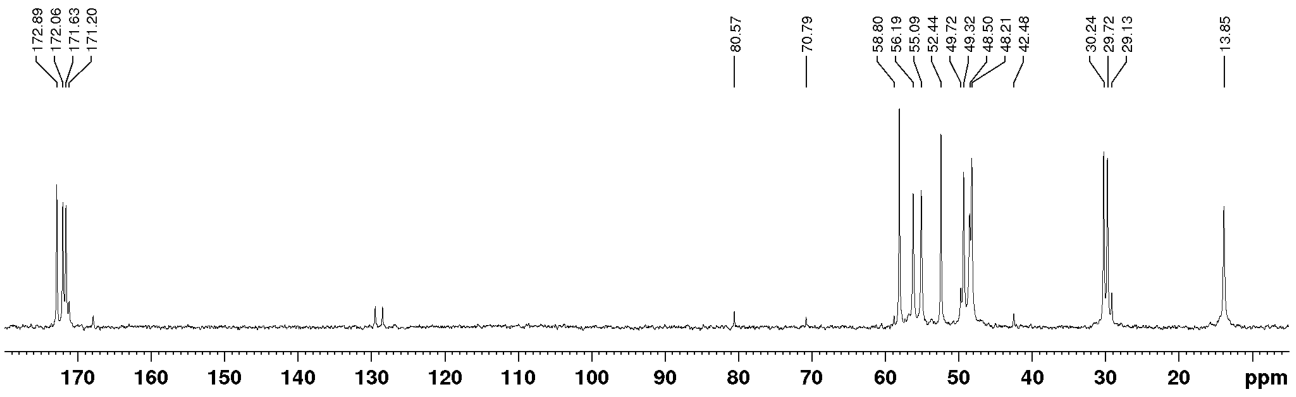

Figure S2: ^{1}H, ^{13}C and ^{1}H-^{13}C hetero-correlated spectra of Man-ISA$_7$ (D_2O)

^{1}H-NMR

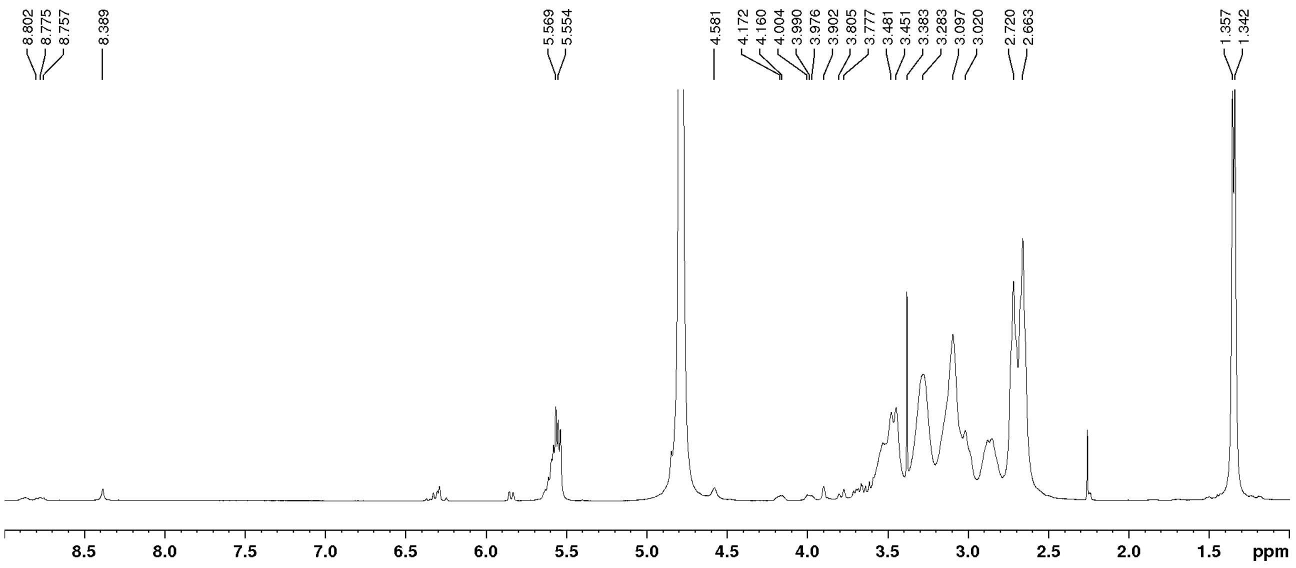

^{13}C-NMR

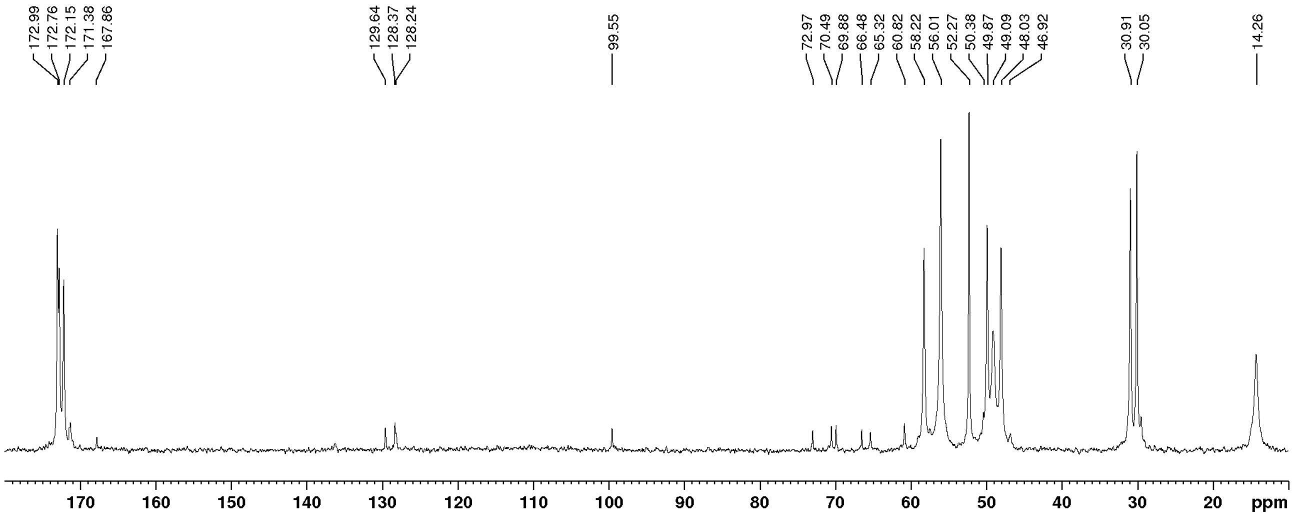

HSQC

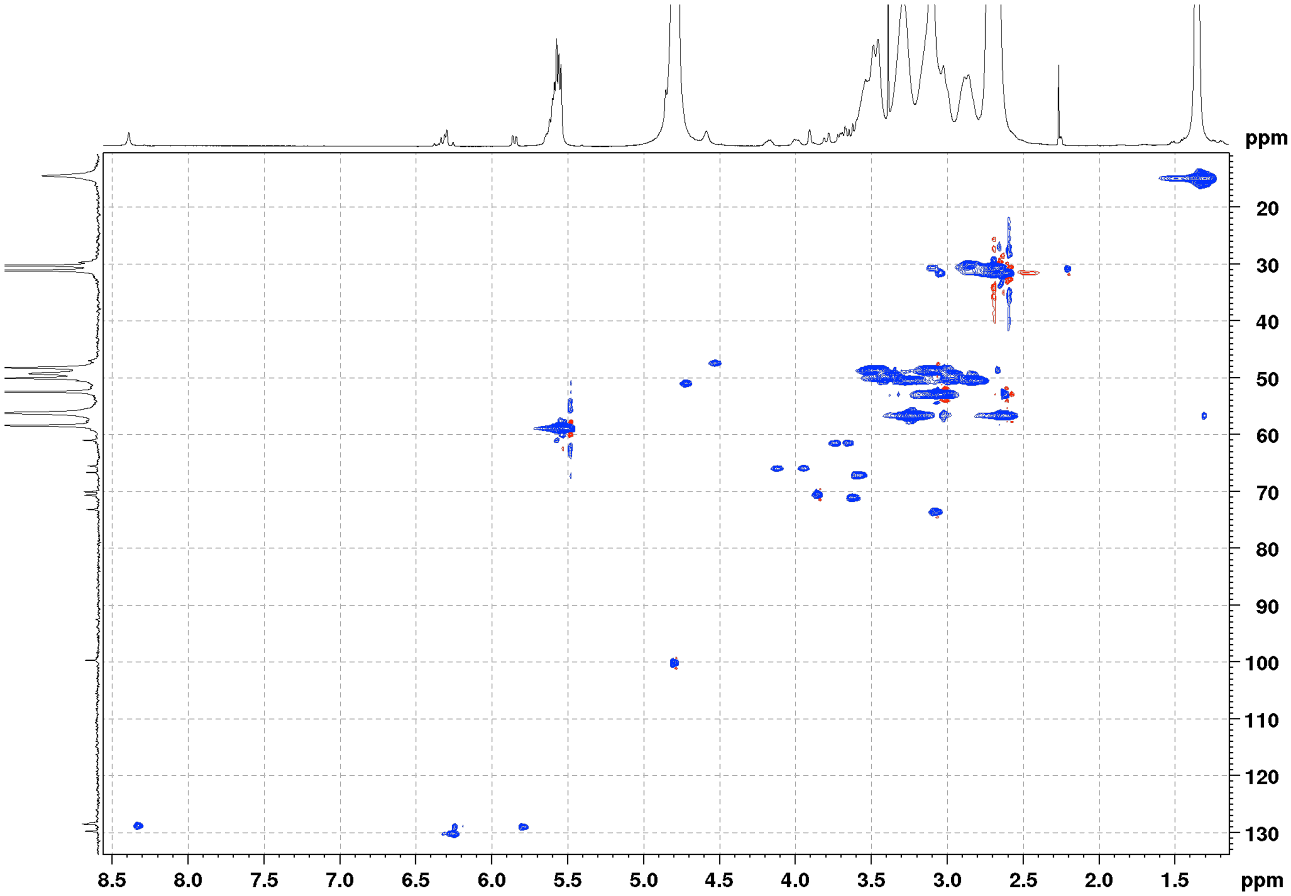
ppm
20
30
40
50
60
70
80
90
100
110
120
130
8.5
8.0
7.5
7.0
6.5
6.0
5.5
5.0
4.5
4.0
3.5
3.0
2.5
2.0
1.5
ppm

Figure S3: ^{1}H, ^{13}C and ^{1}H-^{13}C hetero-correlated spectra of P-AGMA$_{6.5}$ (D_2O)

^{1}H-NMR

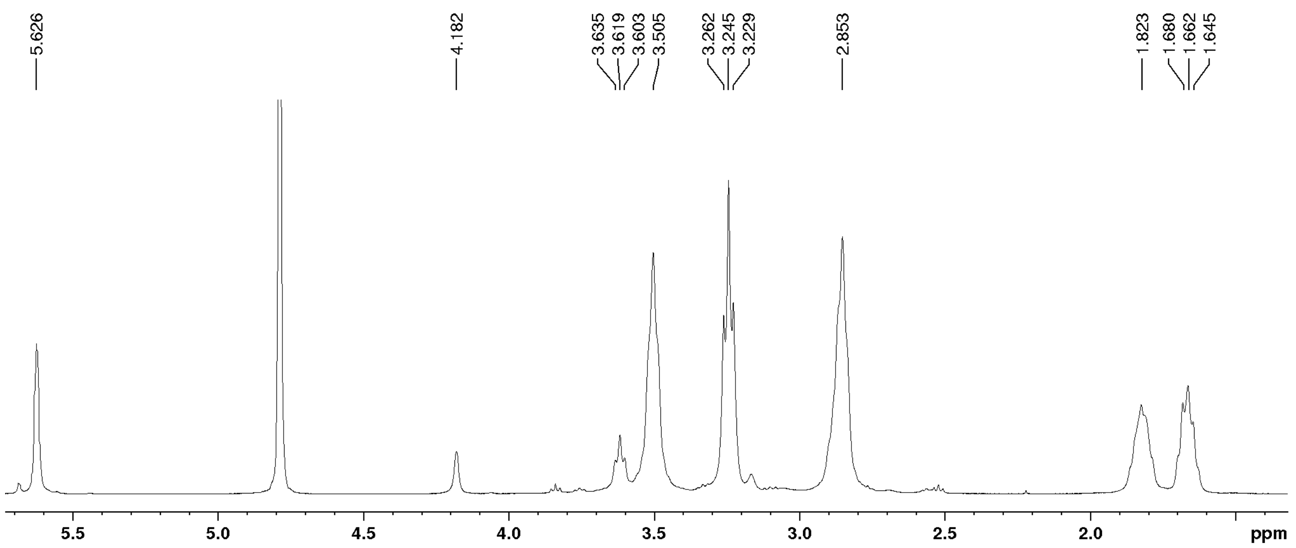

^{13}C-NMR

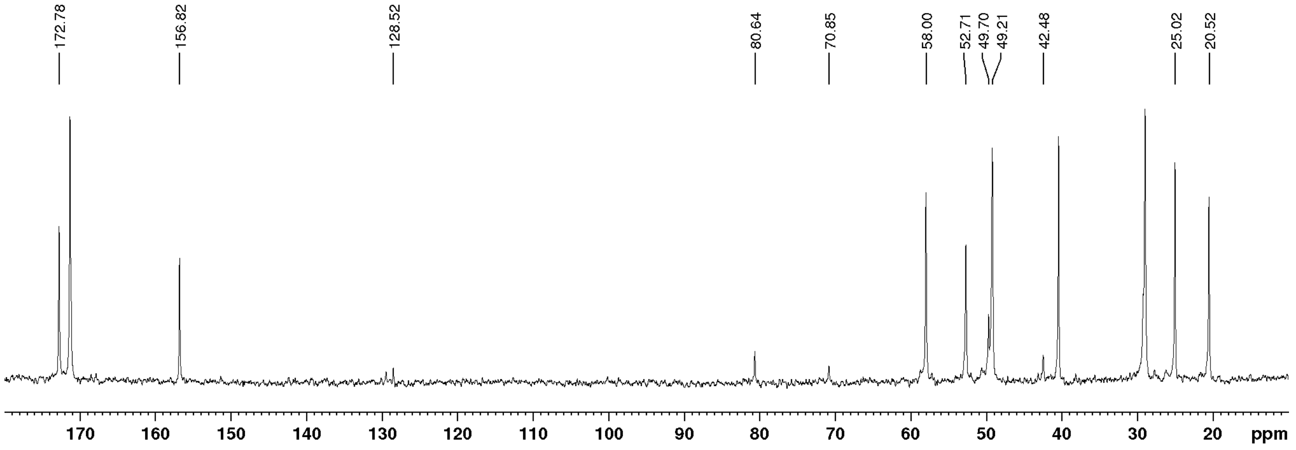

HSQC

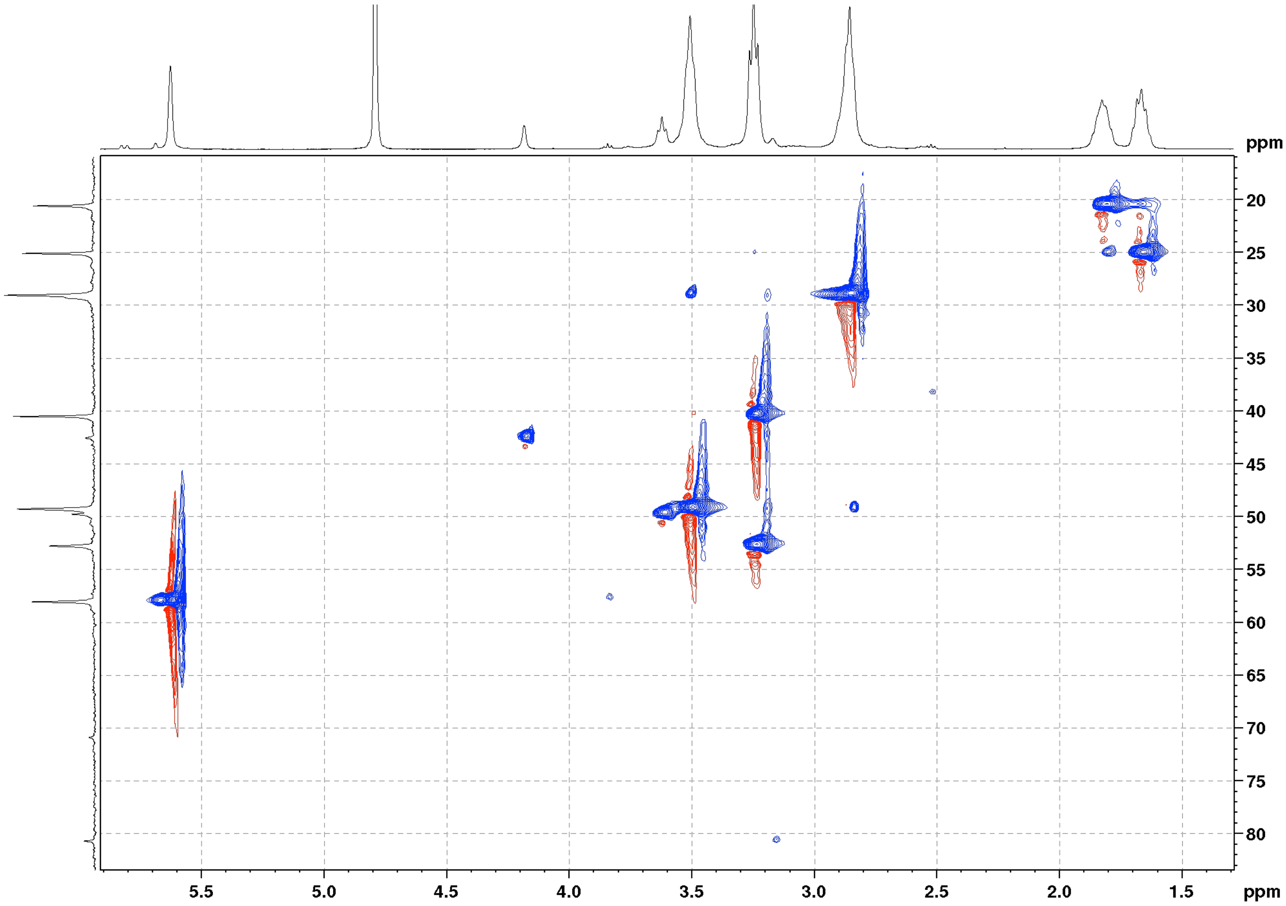

ppm
5.5
5.0
4.5
4.0
3.5
3.0
2.5
2.0
1.5
ppm
20
25
30
35
40
45
50
55
60
65
70
75
80

Figure S4: ^{1}H, ^{13}C and ^{1}H-^{13}C hetero-correlated spectra of Man-AGMA$_{6.5}$ (D_2O)

^{1}H-NMR

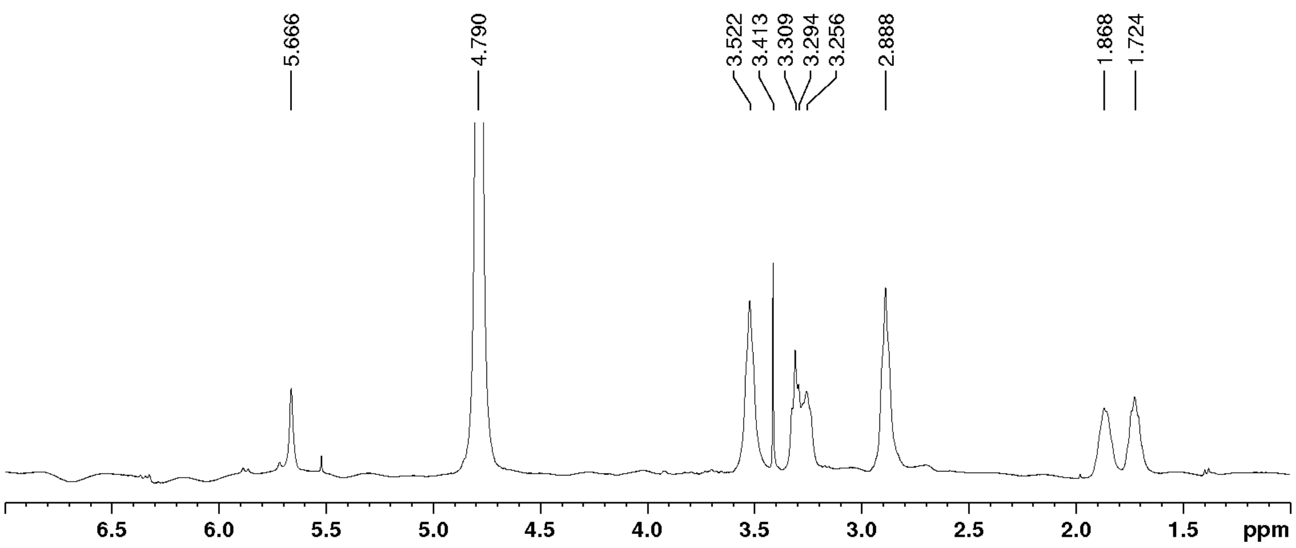

^{13}C-NMR

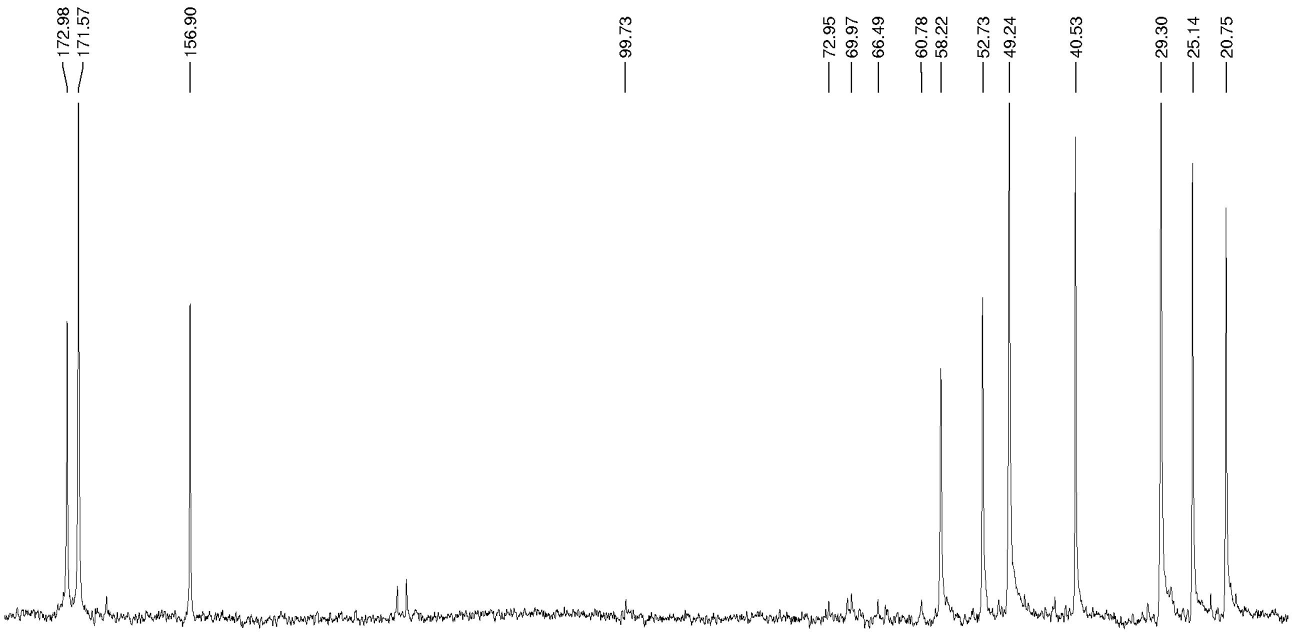

HSQC

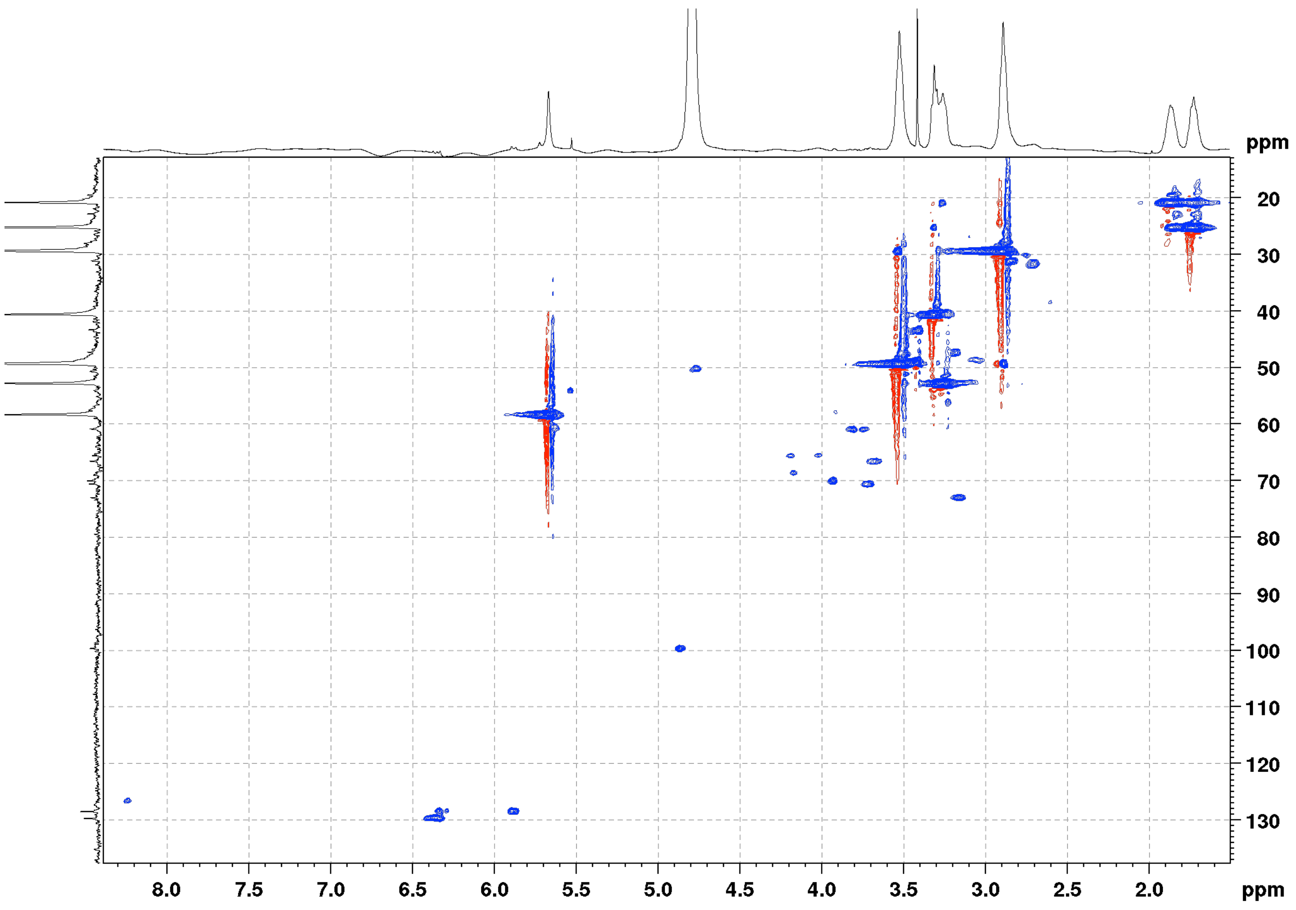
ppm
20
30
40
50
60
70
80
90
100
110
120
130
8.0
7.5
7.0
6.5
6.0
5.5
5.0
4.5
4.0
3.5
3.0
2.5
2.0
ppm

Figure S5. Cytotoxicity of mannosylated ISA23 and AGMA1 on B-THP1/DC-SIGN cells.

Viability of B-THP1/DC-SIGN cells after 3 h and 30 min, 24 h, and 72 h of treatment with different mannosylated unit concentrations was evaluated by staining with 7-AAD, which labels non-viable cells. Experiments were performed in duplicate. Values represent the mean ± SD. Concentrations are referred to the mannosylated unit.

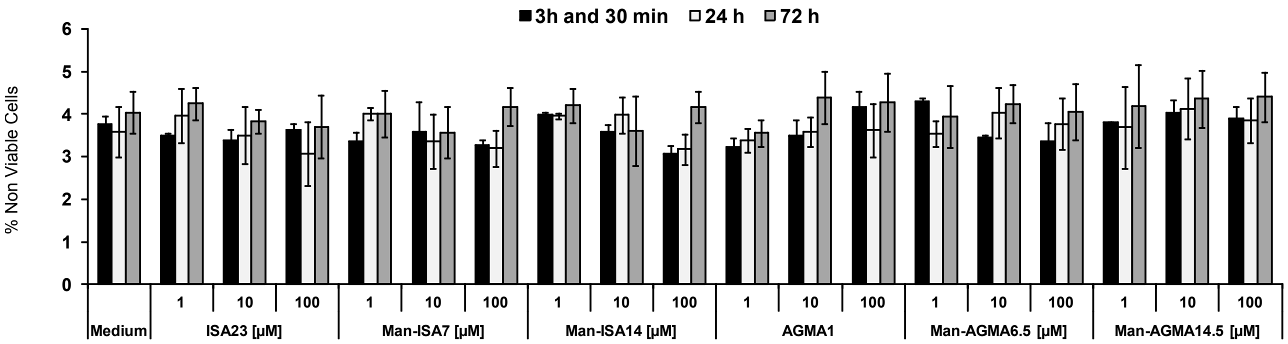

Figure S6. Cytotoxicity of mannosylated ISA23 and AGMA1 on HeLa cells (HPV Assay).

Viability of HeLa cells after 72h of treatment with different compounds was evaluated by MTS assay according to manufacturer instructions. The incubation conditions recapitulate the HPV antiviral assay. Experiments were performed in duplicate. Values represent the mean ± SD. Concentrations are referred to the mannosylated unit.

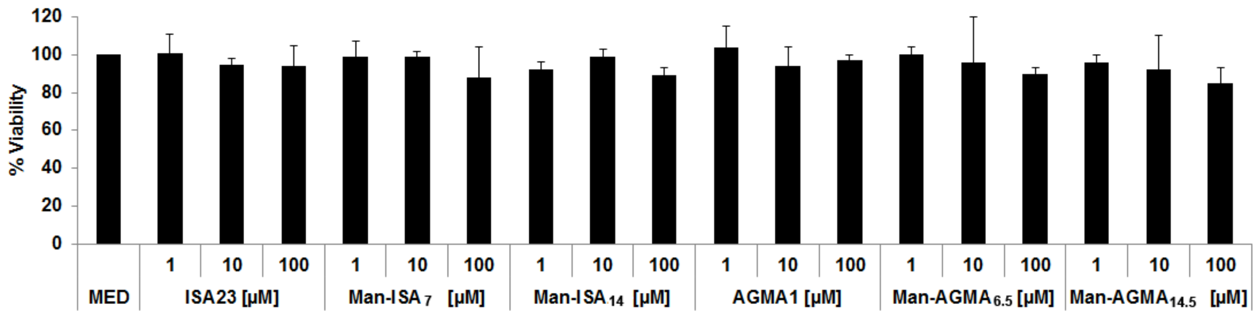

Figure S7. Cytotoxicity of mannosylated ISA23 and AGMA1 on Vero cells (HSV assay).

Viability of Vero cells after 24h of treatment with different compounds was evaluated by MTS assay according to manufacturer instructions. The incubation conditions recapitulate the HSV-2 antiviral assay. Experiments were performed in duplicate. Values represent the mean ± SD. Concentrations are referred to the mannosylated unit.

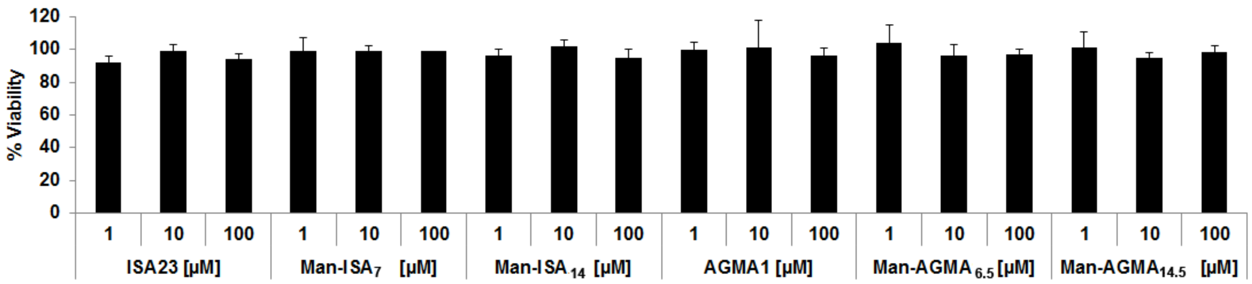

References

(1) Ferruti, P. *et al.* Synthesis, characterisation and antitumour activity of platinum(II) complexes of novel functionalised poly(amido amine)s. *Macromol. Chem. Phys.* **1999**, *200* (7), 1644-1654.

(2) Fulmer, G. R. *et al.* NMR Chemical Shifts of Trace Impurities: Common Laboratory Solvents, Organics, and Gases in Deuterated Solvents Relevant to the Organometallic Chemist. *Organometallics* **2010**, *29* (9), 2176-2179.

(3) Sattin, S. *et al.* Inhibition of DC-SIGN-Mediated HIV Infection by a Linear Trimannoside Mimic in a Tetravalent Presentation. *ACS Chem. Biol.* **2010**, *5* (3), 301-312.
